# Proceedings of the National Conference on Emerging Foodborne Pathogens: Implications and Control. Alexandria, Virginia, March 24-26, 1997.

**DOI:** 10.3201/eid0304.970401

**Published:** 1997


### About the National Conference on Emerging Foodborne Pathogens: Implications and Control*

Infectious diseases transmitted by foods have become a major public health concern in recent years. Response by both the food industry and public health and food safety regulatory agencies to new microbiologic health threats and reemerging pathogens in foods has been primarily reactive. The multiplicity of factors and complex interactions involved in the emergence and reemergence of microbial foodborne hazards and the need for a multifaceted and integrated approach to protecting the population prompted a national Conference on Emerging Foodborne Pathogens: Implications and Control (March 24-26, 1997, Alexandria, Virginia).

The conference, attended by more than 400 scientists in basic and applied research, epidemiology, and public health, was organized to elucidate programs and initiatives that could be used to identify and respond appropriately and proactively to emerging and reemerging foodborne disease threats.

Representing the conference organizers were Dr. Alex Malaspina, president of the International Life Sciences Institute; Dr. David Satcher, director of the Centers for Disease Control and Prevention; Mr. Thomas Billy, administrator of the Food Safety and Inspection Service of the U.S. Department of Agriculture; Dr. Fred Shank, director of the Center for Food Safety and Applied Nutrition in the U.S. Food and Drug Administration; and Sir George Alleyne, director of the Pan American Health Organization. Their opening remarks reflected a strong commitment to collaboration among different sectors, development of integrated approaches to food safety, implementation of President Clinton's food safety initiative, and international cooperation in the fight against foodborne disease.

Nobel laureate Dr. Joshua Lederberg delivered the keynote address, in which he called for a global public health approach to the threats posed by microbial foodborne illness. Dr. Richard Hall's closing address summarized the key points of the conference presentations, emphasized the need for concerted control efforts by the public and private sectors, and suggested prioritizing foodborne disease risks according to their probable impact.

Conference organizers hope that the publication of conference presentations and discussions in this journal will stimulate initiatives to improve the safety of food and draw much needed attention to foodborne microbial hazards.

---

*The conference was sponsored by the International Life Sciences Institute (ILSI), ILSI North America Technical Committee on Food Microbiology, the Centers for Disease Control and Prevention, the U.S. Department of Agriculture, and the U.S. Food and Drug Administration, in cooperation with the Food and Agriculture Organization of the United Nations and the Pan American Health Organization/World Health Organization. Conference grant support was also provided by the National Institute of Diabetes and Digestive and Kidney Diseases. Additional support was provided through unrestricted education grants from the American Meat Institute, American Society for Microbiology, American Veterinary Medical Association, Animal Health Institute, Association of American Veterinary Medical Colleges, Frito-Lay, Inc., Land O'Lakes, Inc., McDonald's Corporation, National Food Processors Association, The Quaker Oats Company, Roquette America, Inc., and Ross Products Division, Abbott Laboratories. The members of the Technical Committee on Food Microbiology are Campbell Soup Company, The Coca-Cola Company, General Mills, Gerber Products Company, H.J. Heinz Company, Kraft Foods, Inc., Lipton, M&M/Mars, Nabisco, Inc., Nestlé USA, Inc., PepsiCo, Inc., The Pillsbury Company, and The Procter & Gamble Company.

